# Substance availability and use in ex-professional ice hockey enforcers

**DOI:** 10.1038/s41598-022-26806-7

**Published:** 2022-12-23

**Authors:** Michael Gaetz

**Affiliations:** School of Kinesiology, University of the Fraser Valley, Chilliwack, BC Canada

**Keywords:** Psychology, Medical research

## Abstract

Some ex-professional ice hockey enforcers (players whose primary role was fighting) have experienced negative health outcomes following their careers including substance use. Some have suggested that negative post-career outcomes following a career in contact sport relate specifically to neurotrauma. The purpose of this study was to determine whether ex-professional ice hockey enforcers were negatively impacted by substance use during and/or following their careers. It was hypothesised that given their role in the sport, significant exposure to injury (including concussions) occurred, leading to challenges post-career including substance use. This study utilises a mixed methods quantitative and qualitative approach with one-on-one semi-structured interviews and questions related to substance use. This hypothesis for this study was not supported. Participants in this study reported low levels of substance use post-career. Patterns of substance use during career varied by era with a change in use from alcohol and over-the-counter stimulants to opioids, sleep aids, and anabolic androgenic steroids (AAS) estimated to occur near the mid to late 1990s. Four participants described patterns of excessive alcohol use during their careers. Stimulant use was prevalent in ice hockey pre-mid-1990s. The use of prescription opioids and sleep aids was reportedly rare before the mid to late 1990s, but eventually became easily attainable via team medical staff and prescription sharing. Two participants from the later era also reported use of AAS. This sample of ex-professional hockey enforcers experienced a significant number of concussions, continue to have challenges with chronic pain, and were exposed to several unique stressors during their careers, the effects of which may have varied based upon how the role was viewed. A combination of these factors may have resulted in substance use in some of these athletes during, but not following their careers.

## Introduction

There is a longstanding and complex relation between substance use and sport. For example, the scientific literature provides evidence for use of alcohol early in sport participation. For team sport, patterns of excessive and/or regular alcohol use are well-established by the time the athlete enters college or university^1–6^ with binge drinking during high school being a significant predictor of later use^7^. It has been shown that alcohol use associated with sport can begin during the middle and high school years^8^. In their study, Veliz et al.^9^ provide evidence that adolescents who participated in competitive sport had higher odds of alcohol intoxication over a 30-day interval and had an early onset for intoxication (4th–8th grade). The effect was stronger for those who participated in contact sport^9^. In their review, Lisha and Sussman^5^ stated that for male high school and college athletes, ice hockey athletes were the most likely to report high levels of alcohol consumption. This pattern was also supported by the NCAA who in 2018 reported that the highest rates of binge drinking by NCAA athletes occurred in lacrosse and ice hockey^10^. Finally, the dangerous extent to which alcohol can be used by ice hockey athletes and coaches was described in a season long study by Roy and Camiré^11^.

There is also research evidence for stimulant use in sport. For example, the use of stimulants in sports such as American football extends back to the 1940s^12^. Recent trends show that this has not changed, with 85.9% of U.S. college students stating that they used energy drinks, dietary supplements, or prescription medications within the last year to enhance athletic performance^13^. Bents, Tokish, and Goldberg^14^ found that 58% of college ice hockey players admitted to past or present use of stimulants including pseudoephedrine and ephedrine. Over 33% of these athletes stated they would use a banned substance if it improved their chances of playing in the NHL^14^.

Performance enhancing drugs, such as anabolic androgenic steroids (AAS), have received attention of late in Olympic and professional sport. As described by McDuff and Baron^12^, the use of AAS to improve athletic performance dates to the cold war era in sports such as powerlifting. AAS allow athletes to train longer and harder to potentiate increases in muscle size and strength. The benefits are not without serious side-effects. In addition to the physical effects, cognitive-emotional risks associated with AAS include increased hostility, aggression, irritability, mood lability, and major mood syndromes^12^. Visuospatial performance deficits have also been associated with AAS use^15^. AAS use has been reported to be high in recreational athletes^16^. The use of performance enhancing drugs (PEDs) in college athletes has been reported to be relatively low 3.1%, however those who did use PEDs also reported using more alcohol and recreational drugs^17^. Historically, the use of PEDs in college ice hockey has been low^10^. An NAIA sample reported that 0.8% of male student-athletes used AAS and 0.3% used human growth hormone^18^. Finally, in a sample of 33 retired professional ice hockey athletes, one (`3%) had a history of AAS use during his career^19^.

Opioid use has also been reported by athletes in many sports. In an annual sample of approximately 15,000 U.S. high school athletes, nonmedical use of prescription opioids was reported by 8.3% of respondents, with heroin use reported by 0.9%. Both ice hockey and weightlifting were associated with increased odds of past-year heroin use^20^. A review of opioid use in high school athletes suggested lifetime opioid use rates of 28% to 46%^21^. In U.S. college athletes, the percentage of users has dropped slightly from previous years but continues to be relatively high. The NCAA^10^ reports that 11% of student athletes used opioid pain medication with a prescription, three percent used opioid pain medication without a prescription, and two percent reporting misusing opioid pain medication^10^, with lower rates of use reported for the NAIA^21^. Like alcohol use, opioid use was the highest for men’s lacrosse (17%) and ice hockey (13%)^10^. Professional American football players have reported significant levels of opioid use and misuse. Cottler et al.^22^ reported that over half (52%) of retired athletes admitted to using opioids during their National Football League (NFL) career with 71% reporting misuse. Their rate of current opioid use was seven percent which is approximately three times the rate of the general population. The three variables that best predicted current opioid misuse in this sample were significant chronic pain, undiagnosed concussions, and heavy alcohol consumption^22^.

There is a paucity of peer-reviewed science on substance use in professional ice hockey. A study by Esopenko et al.^19^ reported that in 33 ex-professional hockey athletes, six had a psychiatric diagnosis of past alcohol dependence and abuse with one current, two had a history of non-alcoholic substance abuse with one current, with 12% of the sample using opiates at the time of the study. Secondary (non-academic) sources such as autobiographies and biographies on professional ice hockey enforcers (players whose primary role was fighting and protection of “skilled players”) contain ample evidence of substance use during and post-career^23–25^.

The Athlete Post-Career Adjustment (AP-CA) model^26^ was developed to provide a more complete understanding of athlete post-career functioning. The AP-CA model states that four factors may be related to significant negative outcomes following retirement from contact sport: neurotrauma, career transition stress, chronic pain, and substance use. In this model, depression may be present prior to an athletic career or may have developed secondary to any of the model’s elements^26^. A substantial overlap exists between symptom profiles for those with a history of depression, substance use, chronic pain, athlete career transition stress, and CTE symptomology^26^. For example, substance use causes a multitude of behavioural and cognitive-emotional changes that correlate with mental health status and chronic pain and overlap with symptoms of the sequelae of neurotrauma^26^. Therefore, to fully grasp the unique impact that a history of neurotrauma has on post-career functioning, it is essential to understand the substance use patterns within a sport.

The purpose of this study was to determine whether a sample of ex-professional ice hockey enforcers were impacted by substance use during and/or following their careers. A 15-item, semi-structured, in person interview format was utilised with questions intended to elicit responses related to neurotrauma history, chronic pain, career transition stress (athletic identity), substance use and depression consistent with the AP-CA model^26^. It was hypothesised that given their role in the sport, these athletes had a significant risk exposure to concussive injury that could lead to substance use during and post-career.

## Materials and methods

This study employed two forms of mixed methodologies that approach science through an Indigenous knowledge and Western lens. The methodology and philosophical underpinnings in this study have been described previously and the content in this section partly reproduces their wording^27,28^. All methods were carried out in accordance with relevant guidelines and regulations.

### Participants

The participants in this study have been described previously^27,28^. Briefly, a total of 10 retired professional ice hockey enforcers agreed to participate in in-person interviews. Participant age ranged from 31–61 years (mean = 48.9). The range in calendar years for the participant’s careers in professional hockey was from 1978–2014. Two participants had estimated approximately 100 career fights with six reporting over 200 career fights (range 100–250; mean = 218.5).

### Procedure

This study is a part of a broader ongoing research project. In this study, a “hockey fight” was operationally defined as participation in a fight between two players where a five-minute major penalty (In hockey, a major penalty is given for more serious infractions. It differs from a minor penalty in that it is longer (5 or 10 min instead of two) and that the player must remain in the penalty box for the entire duration of the penalty whereas minor penalties end after two minutes *or* when a goal is scored while the player is serving the penalty.) was assessed for “fighting” during games. A fight during practice resembled a fight that occurred during games but with no penalty assessed. Both were included as hockey fights in this study. An “ice hockey enforcer” was operationally defined as a professional ice hockey player whose role on the team included bareknuckle fighting in a hockey game or practice, was recognized by other enforcers as someone who had this role and had a minimum of 100 fights total in amateur and professional hockey. A “professional” was defined as a player who had signed a professional contract with a hockey organisation and had played professionally for at least one season. Participant recruitment was initially done by convenience sampling with snowball sampling utilised once the contact information of ice hockey enforcers who were willing to participate in the study was provided. A total of 15 ex-professional ice hockey enforcers were invited to participate with 10 agreeing. The reasons provided for declining to be involved in the study included time conflicts associated with the role of hockey scout, being concerned about how involvement in the study would affect their careers within professional hockey or perceiving a conflict between the purpose of the study and having a leadership role within a professional hockey alumni association. The ethics of the research were reviewed and approved by the University Human Research Ethics Board of the University of the Fraser Valley. Written informed consent was obtained from all participants. The informed consent process occurred immediately prior to the interview and included a review of the informed consent letter, an opportunity for questions and clarification, and was followed by written informed consent.

The interview guide was developed to elicit conversation that encompassed the issues surrounding concussions in ice hockey as well as other factors that may affect post-career functioning^29^. The one-on-one semi-structured interviews were composed of 15 questions and follow-up prompts. The interview was premised with a general statement that the purpose of the study was to allow participants to provide their perspective about their role in ice hockey and the potential negative outcomes that they have heard about in the media and biographical accounts. This signalled to the participants that they were in a position of expertise and power regarding the narrative. Two questions (with follow-ups) were specific to substance use: (1) “During your playing career, how often did you take medication to help you manage pain, sleep, or mood? Follow-up: Did you continue to take these medications post-career?” and (2) “Were performance enhancers used by anyone that you knew in hockey (e.g. Effedra, amphetamines, steroids, andro, HGH)? Follow-up: Have you ever used any of these performance enhancers? If so, when, how often, and when was the last time you used them?” The duration of the interviews was between 25–99 min (mean = 60.9). It was stressed that the information provided would be kept confidential and that any content that would link a response to a specific participant would be anonymised.

Interviews were audio-recorded using both a Panasonic RR-US551 recorder and a Samsung Galaxy 4 smartphone. The audio file on the RR-US551 recorder was deleted following the interview once the smartphone audio file was verified as complete. The digital audio files were then transferred to a password protected secure laptop and the original on the smartphone was then deleted. The audio files were subsequently transcribed verbatim by the researcher or a student research assistant. Once transcription for each file was complete, the transcripts were sent to the personal email of the participants for review and editing. Once the transcript was approved, the digital audio file was deleted from the password protected computer. Non-descriptive labels were used for each participant (e.g. P1). Player, city, and team names were anonymised (e.g. *NHL City*).

### Data analysis

**Figure 1:**
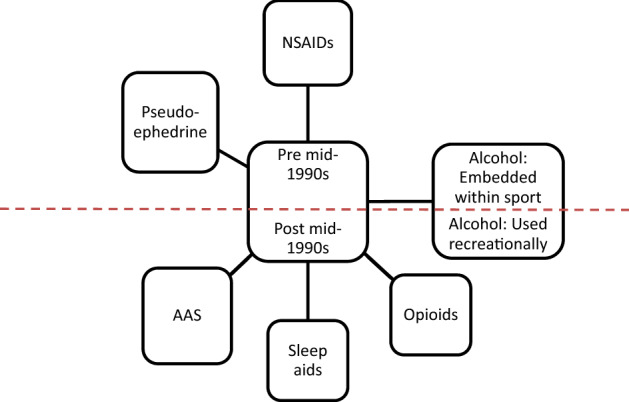
Patterns of substance use by era. During the era pre mid-1990s, substances such as alcohol, non-steroidal anti-inflammatories (NSAIDs), and pseudoephedrine were prevalent in dressing rooms and during travel. During the era post mid-1990s, substances like opioids and sleep aids were frequently used as were anabolic–androgenic steroids (AAS).

The US National Institutes of Health (NIH) has recognised mixed methods research as beneficial for the comprehensive understanding of health issues^30^. The strategy for the use of mixed methods in this study was to initially quantify the nature and frequency of substance use into descriptive themes within empirically derived primary categories (Table 1). This quantitative frequency analysis transformed qualitative responses from interview transcripts into substance use variables^31^. Therefore, the descriptive themes provided a contextual basis for the qualitative thematic development to follow^32^. In some cases, qualitative analysis involved the development of interpretive themes based on the descriptive themes (Table 1; Fig. 1). The method used for thematic analysis was that of Braun, Clarke, and Weate^33^. The development of descriptive quantitative themes followed by more substantial interpretive themes is consistent with an explanatory sequential design where the qualitative component is employed to explain or contextualize the earlier quantitative results^31,34–37^.

**Table 1:** Thematic organisation based on comments associated with substance use patterns as well as descriptive and interpretive themes. Frequencies for descriptive themes are shown in Fig. 1.

Category	Descriptive themes	Interpretive themes
Beverage Alcohol	Alcohol use	Embedded within the culture
		Embedded within the sport
		Substance availability and era: *pre* mid-1990s*
Prescription and non-prescribed substances	NSAIDs	Substance availability and era: *pre* mid-1990s*
	Pseudoephedrine	
	Opioids	Substance availability and era: *post* mid-1990s
	Sleep aids	
	AAS	
	Illicit substances	

Asterisk* indicates an era where the substance was prevalent for use in the dressing rooms on during team travel. NSAIDS = non-steroidal anti-inflammatory medications; AAS = androgenic anabolic steroids.

### Ethics approval and consent to participate

Ethical approval and the consent process are described on page 11. The ethics of this study were reviewed by the Human Research Ethics Board of the University of the Fraser Valley. The ethics reference number is: 1067K-18.

## Results

### Substance availability and career era

Participants in this study played professional ice hockey during a 36-year span from 1978–2014. It became evident that the patterns of substance use for players who played in the 70’s and 80’s were not the same as for players in the late 90’s or post-2000 (please refer to Fig. 1). This pattern of divergence was observed for alcohol use as well as prescription, non-prescription, and illicit substances. The mid-1990s seemed to be when the transition in the patterns of substance use occurred.

### Alcohol use

#### Embedded within the culture

Six participants described how alcohol was present in their culture. Comments such as ‘I don’t have a problem with alcohol though I can consume a lot of it at times … like every other Canadian’ (P5) were relatively common. Two participants described alcoholism in their immediate families. P4 shared ‘I come from a long line of drinkers. …my grandfather died of sclerosis of the liver. My Dad died of sclerosis of the liver.’ (P4). Another participant stated:

‘My mom’s an alcoholic. My dad probably was an alcoholic at a certain time …when we were younger he’s the one that did all the drinking and my mom kind of took over the bottle and she’s the alcoholic still. …my grandparents on her side are both alcoholics.’ (P9)

#### Embedded within the sport

Six participants commented on how drinking alcohol was a part of their experience as ice hockey players. Having access to beer in the dressing room or while traveling after a game was described by several players. For example, P7 stated “If we had a big win, then the coach would walk by and hand you cans and it was fun.’ The use of alcohol for most started with their experiences in junior hockey:

‘booze is certainly engrained in the culture of hockey, at a young age, I mean you move away from home and, they’re hardly legal to drive half these guys and they’re boozing and you know what I mean. They’re legal to drink? They’re certainly not, half of them, um, it’s a problem…’ (P6).

At some point during the 1990s, the environment regarding alcohol availability in dressing rooms began to change. Three participants commented on how alcohol use in the dressing rooms was eventually phased out. P10 stated:

‘…when I came in with *NHL city*, the game was over, there was a bucket of beer. You had a couple beers, you spent 30 minutes maybe 45 talking to your teammates… now they come in, they shower and do their interviews and they (expletive) off with their girlfriend or wife, you know?’

P9 described the transition from beer to the availability of blended non-alcoholic drinks to aid in post-game recovery:

‘And by the end it was blenders, you know what I mean? …you know *NHL player* and I would be sitting there in, *NHL city*… and we talked about how before you hear a (*beer opening sound*) like the, like the crack of a bottle… Now you hear the blender’ (P9).

#### Excessive Alcohol Use

Four participants described patterns of excessive alcohol use that occurred during their professional careers. P4 shared ‘Sometimes I didn’t know when to say when… I never drank hard alcohol… I was always just pretty much strict beer drinker’ Another commented ‘…uh did I drink? Ya, I drank a lot. I’ll be honest with you.’ (P8). One participant described his pattern of regular alcohol consumption this way:

‘After practice you went for lunch and had 10 beer. Then you’d go home and have dinner. …and if you didn’t play for three to four days, guess what? Your lunch turned into an all-night affair … I learned to be a good drinker through the NHL’ (P9).

Two participants described substantial alcohol use but in combination with other substances. For one participant, the alcohol became part of a life of excess that coincided with other changes in the sport at that time. P6 described that his alcohol use was:

‘not every day, but when I partied, I partied hard, like but it was still a big part of the party in my life (*emphasis*) …you know it didn’t matter how hung-over I was the next day, I was fighting, fighting the biggest guys, I played hard you know what I mean? So I think that between the fighting and managing (*clears throat*) you know the fight and the anxiety of the fight a lot of the times with alcohol… it became probably a little bit too extreme’.

Near the end of his career, P9 described the combined challenges of injury, end of career transition, and substance use:

‘…I was a runaway in *NHL city* because, you get injured - what (do) guys do, you get hurt… and you pull yourself away from the team so you don’t feel a part of it. And it’s a hard pill to swallow when you show up at the rink and everybody’s getting ready for the game and you’re just a (expletive), you’re sitting, you’re not a part of it. So, where do you feel a part of it? Well you go drink with your friends there, right? And the girls and …the clubs whatever …in *NHL city* I went to a whole nother level of drinking.’

### Prescription and non-prescribed substances

#### Pre mid-1990s: Pseudoephedrine and NSAIDs

Seven participants who played professional hockey before the mid-90’s generally denied regular use of prescription pain relievers and instead relied primarily on non-steroidal anti-inflammatory (NSAID) medication to manage pain. Comments such as ‘I’m hearing stories and I can’t relate to it …I was …pretty active in fighting …and I probably did have a lot of pains or aches or pains or whatever but I never took a medication of any type’ (P3) and ‘Probably the only medication we took would have been (*laughs*), like I say, tape an aspirin to it’ (P8) were common. Another participant commented that:

‘the team doctor used to give it, it was an anti-inflammatory …it was like they had the tester bottles... So you could get them every day if you wanted right? …so I mean I used to live on (them) …because it was like you’d just, it would make your body feel good right?’ (P4).

At times, pain management included alcohol: ‘And of course they also hand you a six pack of beer in those days …a strip of pills and a six pack of beers and go to the back of the bus and uh wake up in Tulsa’ (P7). Two of the participants mentioned the regular use of cortisone injections by the medical staff of one NHL team. Another participant mentioned the regular use of Toradol (an injectable NSAID).

When asked about PEDs, seven participants had first-hand knowledge of pseudoephedrine use on their teams. For example:

‘…we took Sudafed right? Sudafed, especially if it was a big game and you knew you might be, you knew you had to fight *NHL enforcer*, or (*laughing*) guys like that …it was like, you had a couple of Sudies in you so that you were pumped up and ready, you were revved up.’ (P4).

P7 comments on the transition to the common use of pseudoephedrine near the end of his career:

‘what came at the tail end (of his career) was Sudafed, that was the big thing …guys were starting to realize (it) could pick you up just before the game so there would be the green mint flavour Sudafeds in the bathroom’.

Another participant stated: ‘in the room, you would have your gum, you’d have your laces, you’d have your tape, you would have a thing of Sudafed’ (P8). While some participants admitted to use of Sudafed, others reported not liking the effects or not needing it to be effective in their role. These participants had opinions such as ‘I honestly tried it a few times but, I didn’t really, didn’t really need it’ (P5) and ‘did I ever take any of that? No because I didn’t need it. A cup of coffee was fine’ (P8).

When asked about AAS, seven participants who played prior to the mid-1990s generally denied use and/or did not have first-hand knowledge about use. A typical comment was ‘they talk about steroids and stuff, I didn’t see a lot of that in our game. I didn’t see it.’ (P5). Other participants from this era mentioned rumours or suspicions of AAS use but had no definitive first-hand knowledge. Additionally, the response was similar for the use of sleep aids. Participants understood why they may be considered beneficial for management of sleep when traveling through different time zones or when in pain, but none of the seven reported first-hand knowledge of use. P3’s comments were representative of participants from this era: ‘I don’t want to try something just to say oh this will make you feel good, or here’s an upper, or, you know I can’t sleep, give me some medication. I’m just, I’ve never done that’.

#### Post mid-1990s: Opioids, sleeps aids, and AAS

For the three participants whose careers extended beyond the mid-1990’s, the patterns of substance use changed dramatically. Alcohol and pseudoephedrine were no longer provided by teams and/or present in dressing rooms. The most significant change was the prevalence of prescription opioids and sleep aids that were available from the team medical staff or via medication sharing. Typically, participants who used opioids also used sleep aids. For example, P1 stated:

‘I would take sleeping pills on the buses and stuff. Ambien. And then uh, there was a point… so if I had a legit injury, I would, I would take pain killers… Um, and then there’s a point in… I would say my second last year, something like that where… I was starting to get a little bit more heavy into the pain killers …and, it was probably a little bit of a problem. I was able to, kind of… snap out of it early enough that it didn’t become a huge problem’ (P1).

This participant continued that access to these products was relatively easy:

P1: ‘Uh, those were prescribed out pretty, pretty regularly.

I: Were they prescription or did you get them from…?

P1: They were prescription, well the doctor would write a prescription for you (*clears throat*) and then, you essentially just share them with everybody on the bus’.

P6 described it this way:

‘probably in like the late 80s, 90s is probably when the pill, the pill mill started rolling in. I really got introduced to it in my first-year pro… it was around everywhere. …I started …like you know a 5 mg Percocet here, a 5 mg Percocet here, and there was sleeping pills …when I got with the *NHL team* is when I started flying now, and that’s when I started …to feel the wear and tear so I’d be on the plane and I’d be like, “hi *team trainer* I need a muscle relaxer or you know a sleeping pill” …then you’re out at the party, …you think that a guy’d be smoking a joint or something like that. It’s like oh I got some Percocet 10s, it was, it was just passing them around, eating them and washing them down with, well beer at that time …that was just normal’ (P6).

While P2 stated that he rarely utilised these substances, he saw how their use affected his teammates:

‘But definitely played with guys who were addicted …and when one guy got it, like “hey can I have some, can I have some?” And if you wanted some of your own it was not hard to get. You could say anything… Whether you wanted to sleep… I had guys who were popping two Ambiens a night to sleep now because they were just hooked now, they couldn’t sleep without it’ (P2).

This participant made an interesting observation linked to the summer of 2011 and the deaths of three ice hockey enforcers. He stated: ‘… before all those suicides happened it was just go (to) the doctor, “Hey I got a sore shoulder. There you go.” But then after I think it was Boogaard …they clamped down hard’ (P2). Another participant described how sleep aids were used recreationally by other players:

‘they called it the Ambien Olympics. …we were in *NHL city* and we got a game tomorrow night, they flew in today, they’d go over to some restaurant and they’d order some wine and some food and they’d eat it and towards the end of the meal there would be one guy that wouldn’t, the other guys would all pop a couple Ambien, and they’d call it the Ambien Olympics to see if they could find their way back to the (expletive) hotel’ (P10).

During the later era, participants also had first-hand knowledge of AAS. Two participants reported using AAS prior to training camp to add weight and gain strength with the hope of improving their chances for a roster spot on a team. P1 shared: ‘…it was pills, it was, uh, Dianebol… D-Bol. I did one cycle. I guess you could call it one bottle for a month’ (P1). P6 added: ‘I was doing steroids even before that, from 18–22, you know, did six to seven cycles of steroids in that amount time. In juniors ya … year, and then ah, my first couple years pro, but it was my, it was in the off-season’ (P6). Both participants mentioned that they believed the use of AAS did not help them significantly in their ice hockey careers.

### Illicit substances

One participant described the use of cocaine late in his career and into retirement. His use of cocaine co-occurred with what he believed was an alcohol addiction. A different participant, while not taking cocaine himself, shared: ‘it’s such a no–no now for these kids to come in and even smell like a little bit of beer. So, they (expletive) do the pills, they snort coke, like cocaine’s (making) a huge (expletive) comeback in the NHL. It’s awful.’ (P10). A third participant has used cannabis in various forms since his teens and at various times in substitution for other substances such as opioids to manage pain. He continues to use cannabinoids to this day and advocates for their potential medicinal benefits.

## Discussion

The purpose of this study was to determine whether the post-career functioning of ex-professional ice hockey enforcers was impacted by substance use during and/or following their careers. It was hypothesised that participants would report substance use post-career; this hypothesis was not supported. However, there were reports by several participants of substance use during and post-career by themselves or their teammates that reflected a culture of substance use that varied over time. The patterns of use varied by era with a change in use from alcohol and over-the-counter stimulants to opioids, sleep aids, and AAS estimated to occur near the mid-1990s. Participants who played prior to the mid-1990s reported that alcohol was often provided by teams following games, but this practice was phased out by the turn of the century. Consistent with research in other sports [e.g.^38^], alcohol use was reported by four participants at some point during their careers with one participant stating that he believed he was an alcoholic. As previously reported^14^, stimulant use was prevalent in the sport pre mid-1990s and stimulants were provided by the teams in most cases. Stimulant provision by teams was eventually phased out. The use of prescription opioid pain medication and sleep aids was reportedly rare before the mid-1990s but eventually became easily attainable via team medical staff and prescription sharing with other teammates. Three participants who played after the mid-1990s admitted to the use of opioids and sleep aids. Consistent with Buckman et al.^17^, these two participants also reported the use of AAS prior to training camps with the hope of increasing size and strength to benefit them as enforcers. Fortunately, none of the participants developed prolonged patterns of opioid or sleep aid use that approached the dangerous levels described in biographies and autobiographies of ex-NHL enforcers^23–25^. Finally, one participant reported cocaine use near the end of career that persisted into retirement with another reporting the use of cannabis and cannabinoid products from adolescence to the present.

This study is the first to describe a culture of substance use that is consistent with what has been described in the biographies and autobiographies of some ex-professional ice hockey enforcers. The question as to what caused the high levels of substance use (in particular opioid and sleep aid use) during and post-career that was documented in the biographies of these athletes is unknown, but it has been speculated by some that it is specifically linked to neurotrauma sustained during their careers [e.g.^25^]. However, in these biographies, the use of alcohol, opioids, and sleep aids appears to have initially coincided with fighting-related injuries (e.g. to the hands, shoulders, and back). Similarly, fighting related injuries were previously described in this sample^27^ and caused substantial chronic pain, especially during the ice hockey season. Further, the current sample has previously reported stressors unique to their role as enforcers such as preparation to fight and expendability based on performance^28^. The combination of pain and stressors has led to increased substance use in other athlete populations. Didymus and Backhouse^26^ reported that various substances were used to cope with both injury and competition-related stressors in a sample of rugby athletes. Similar patterns have been described in retired NFL athletes who reported use of prescription opioids to reduce pain, stress, and anxiety^39^. A study by Mannes et al.^40^ reported that among NFL retirees, opioid use that occurred early in retirement predicted not only current use but substantial effects on mental health including moderate-severe depression symptoms nine-years post-retirement. Importantly, a history of concussion can be predictive of increased substance use, especially when combined with mental health challenges^41^. This new evidence is important because of the known links between chronic pain, opioid and sleep aids (not to mention PED) use, and mood disorders [e.g.^42,43^]—all of which are associated with suicide in athletes^44^.

This study had several limitations. The interview content was based on the retrospective recall of participants and therefore may have inaccuracies due to the passage of time. The subject material was focused on substance use. Some participants may not have felt comfortable revealing personal experiences with substance use or what they had observed with other players. In addition, some participants may have limited the frankness of their responses because they continue to work in a hockey-related field and were concerned about how participation might affect employment. This was addressed by removing personal identifiers and allowing participants to edit their transcript for content. Finally, the sample size in this study was 10 participants which could be considered low. However, when assessing sample size, one should also consider the size of the population that the results are intended to generalize to. Based on the operational definition of “enforcer” for this study, the population of interest would likely be in the 100 s. Therefore, when the size of the “enforcer” population is considered, the number of participants in the present study seems more reasonable.

## Conclusions

In summary, there has been much speculation about the post-career functioning of ex-professional ice hockey enforcers. Providing a voice for those who had this role is important because their lived experiences are crucial for an understanding about why negative outcomes have occurred in the past. Previous studies from other sports have shown that substances have been used to cope with injury and stressors related to competition in rugby^26^ and NFL athletes^39^. Participants from the present sample have previously reported a history of concussion^27^, other significant injuries during their career^27^, as well as unique stressors associated with the role of enforcer^28^. It is possible that their patterns of substance use during their career may have been related to some of these factors. Perhaps more importantly, the responses of the participants expose the cultural contexts and risks of substance use that were present during the era that they played. Given that these patterns were a part of the ice hockey culture, they would have been risk factors for all ice hockey athletes. Future studies should prospectively measure risk factors for negative outcome post-retirement beyond a history of neurotrauma.

## Data Availability

The data that support the findings of this study will be made available upon reasonable request from the corresponding author [MG]. The data are not publicly available because they contain information that could compromise the privacy of research participants. Misuse (intentional or unintentional) could compromise confidentiality and may negatively impact employment of the participants within their profession.
